# Artificial Intelligence in Biomedicine: A Systematic Review from Nanomedicine to Neurology and Hepatology

**DOI:** 10.3390/pharmaceutics17121564

**Published:** 2025-12-04

**Authors:** Diana-Maria Trasca, Pluta Ion Dorin, Sirbulet Carmen, Renata-Maria Varut, Cristina Elena Singer, Kristina Radivojevic, George Alin Stoica

**Affiliations:** 1 Department of Internal Medicine, University of Medicine and Pharmacy of Craiova, 200349 Craiova, Romania; diana.trasca@umfcv.ro; 2 Faculty of Medical and Behavioral Sciences, Constantin Brâncuși University of Târgu Jiu, 210185 Târgu Jiu, Romania; dorin.pluta@e-ucb.ro; 3 Department of Anatomy, Discipline of Anatomy, University of Medicine and Pharmacy of Craiova, 200349 Craiova, Romania; 4 Research Methodology Department, Faculty of Pharmacy, University of Medicine and Pharmacy of Craiova, 200349 Craiova, Romania; kristinaradivojevic03@gmail.com; 5 Department of Mother and Baby, University of Medicine and Pharmacy of Craiova, 200349 Craiova, Romania; cristina.singer@umfcv.ro; 6 Department of Pediatric Surgery, Faculty of Medicine, University of Medicine and Pharmacy of Craiova, 200349 Craiova, Romania; alin.stoica@umfcv.ro

**Keywords:** artificial intelligence, machine learning, deep learning, nanomedicine, oncology, cardiology, neurodegenerative diseases, Parkinson’s disease, Alzheimer’s disease, hepatology, medical imaging, robotic surgery, personalized medicine

## Abstract

**Background/Objectives:** This review evaluates the expanding contributions of artificial intelligence (AI) across biomedicine, focusing on cancer therapy and nanomedicine, cardiology and medical imaging, neurodegenerative disorders, and liver disease. Core AI concepts (machine learning, deep learning, artificial neural networks, model training/validation, and explainability) are introduced to frame application domains. **Methods:** A systematic search of major biomedical databases (2010–2025) identified English-language original studies on AI in these four areas; 203 articles meeting PRISMA 2020 criteria were included in a qualitative synthesis. **Results:** In oncology and nanomedicine, AI-driven methods expedite nanocarrier design, predict biodistribution and treatment response, and enable nanoparticle-enhanced monitoring. In cardiology, algorithms enhance ECG interpretation, coronary calcium scoring, automated image segmentation, and noninvasive FFR estimation. For neurological disease, multimodal AI models integrate imaging and biomarker data to improve early detection and patient stratification. In hepatology, AI supports digital histopathology, augments intraoperative robotics, and refines transplant wait-list prioritization. Common obstacles are highlighted, including data heterogeneity, lack of standardized acquisition protocols, model transparency, and the scarcity of prospective multicenter validation. **Conclusions:** AI is emerging as a practical enabler across these biomedical fields, but its safe and equitable use requires harmonized data, rigorous multicentre validation, and more transparent models to ensure clinical benefit while minimizing bias.

## 1. Introduction

The conceptual roots of Artificial Intelligence (AI) can be traced back to the mid-20th century, when pioneers such as Alan Turing and John McCarthy proposed the idea of machines capable of simulating human reasoning and learning. The early decades (1950s–1970s) were characterized by symbolic AI and rule-based expert systems, which relied on manually encoded logic. The 1980s and 1990s saw the emergence of statistical learning and neural network models, marking a shift toward data-driven computation. The explosion of digital data and computational power in the 2010s catalyzed the deep learning revolution, leading to the development of multilayer neural architectures capable of high-dimensional pattern recognition. More recently, transformer-based and generative models have further advanced the field, enabling large-scale, context-aware systems with unprecedented performance in medical imaging, drug discovery, and natural language processing [1,2,3]. AI represents a frontier field within computer science, dedicated to developing systems capable of replicating human intelligence and performing tasks traditionally dependent on human cognition [4]. The primary objective of AI is to imitate and automate complex cognitive functions such as learning, perception, reasoning, and problem-solving. Within this broad domain, methodologies like machine learning, computer vision, robotics, neural networks, and natural language processing play integral roles [5,6].

In medicine, AI-driven tools assist in predicting disease progression, interpreting medical images, and accelerating drug development [7,8].

**Figure 1:**
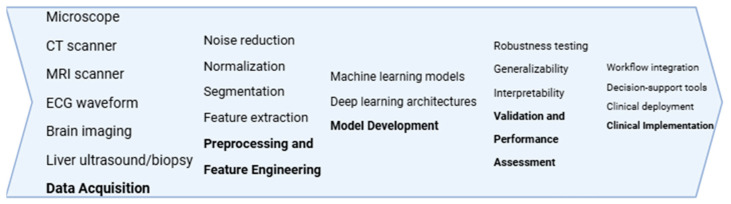
Conceptual workflow underlying AI methodologies in biomedicine, summarizing the progression from multimodal data acquisition through preprocessing and feature engineering, model development, validation and performance assessment, to clinical implementation and integration into healthcare workflows.

The biomedical research community has shown growing interest in artificial intelligence due to its ability to process complex, large-scale biomedical datasets, generate accurate diagnostic insights, and optimize therapeutic decision-making. Within medicine, AI-driven systems contribute to precision diagnostics, image analysis, drug discovery, and robotic-assisted interventions. Their reliability depends on principles such as transparency, interpretability, and data integrity, which ensure trustworthy and reproducible results. Although modern AI techniques have rapidly evolved, their conceptual foundations, rooted in early computational and cognitive science research, continue to inform present-day biomedical innovation [9,10,11,12]. A schematic overview of the core AI workflow in biomedicine, from data acquisition to clinical implementation, is presented in Figure 1.

AI-enhanced systems are also transforming personalized medicine by utilizing patient histories and medical records to determine individualized treatment plans and optimal medication regimens. Data from wearable monitoring devices, which capture heart rates and physical activity, can be integrated into healthcare databases for real-time analysis. With the influx of patient-specific information from diverse sources, AI identifies anomalies, predicts potential medical crises, and alerts healthcare professionals. For instance, hospitals in countries such as Denmark and Norway have adopted AI-based analytic tools to detect inefficiencies and minimize treatment errors within healthcare systems [13,14,15]. Furthermore, surgical robots trained through AI can analyze extensive procedural data to refine surgical techniques, allowing for greater precision and reduced unintended movement [16,17,18]. Beyond spinal procedures, AI is increasingly applied to minimally invasive and robot-assisted surgeries, as well as postoperative monitoring, including the estimation of recovery durations [19,20].

The four domains covered in this review, nanomedicine, cardiology, neurology, and hepatology, were deliberately chosen to represent complementary biological and clinical scales at which artificial intelligence exerts measurable impact. Nanomedicine illustrates AI-driven design and optimization at the molecular and subcellular level; cardiology represents organ-level imaging and physiological monitoring; neurology demonstrates multimodal integration across imaging, electrophysiology, and digital biomarkers; and hepatology exemplifies AI applications in pathology, surgery, and transplant decision-making. Together, these fields provide a coherent framework spanning molecular, structural, functional, and systemic dimensions of biomedicine, allowing for the identification of cross-cutting computational principles and translational pathways that would not be evident within a single specialty.

The aim of this systematic review is to comprehensively synthesize and evaluate the current and emerging applications of AI across key biomedical fields, including nanomedicine, cardiology, neurology, and hepatology. Specifically, the study seeks to identify methodological advances and translational opportunities of AI in these domains; analyze the strengths and limitations of existing evidence; and highlight future research and regulatory directions needed for safe, effective, and equitable clinical implementation of AI technologies.

## 2. Methods

A systematic search was carried out to map contemporary applications of artificial intelligence across oncology (including nanomedicine), cardiology and imaging, neurodegenerative disorders, and hepatology. Electronic searches of PubMed/MEDLINE, Scopus, Web of Science, and Google Scholar were performed between January 2010 and June 2025, using combinations of controlled vocabulary and free-text terms such as “artificial intelligence”, “machine learning”, “deep learning”, “neural network”, “explainable AI”, “nanomedicine”, “nanocarrier”, “cancer”, “cardiology”, “CT”, “MRI”, “FFR-CT”, “ECG”, “radiomics”, “Alzheimer”, “Parkinson”, “liver”, “histopathology”, and “transplant”.

Reference lists of retrieved reviews and key articles were manually screened to identify additional relevant records.

Eligible studies included English-language full-text articles (original research, systematic reviews, or narrative reviews) that described AI-based methodologies, clinical or preclinical applications, validation frameworks, or translational implications within the target domains. To account for domain-specific differences in research focus and methodology, tailored inclusion criteria were applied for each biomedical field.

- Nanomedicine: Studies employing AI or machine learning models for nanoparticle design, drug delivery optimization, or nano-bio interface characterization were included.
- Cardiology: Eligible studies focused on AI-assisted diagnosis, risk prediction, or image-based assessment (echocardiography, CT, or MRI) of cardiovascular diseases.
- Neurology: Included studies addressed AI applications in neuroimaging, neurodegenerative diseases (Alzheimer’s, Parkinson’s), or neurological outcome prediction.
- Hepatology: Studies were included if they used AI tools for liver disease diagnosis, fibrosis staging, hepatocellular carcinoma detection, or treatment outcome prediction.

Across all domains, only peer-reviewed original research articles in English were included, while reviews, editorials, case reports, and non-human experimental studies were excluded.

Priority was given to work published within the last five years, while seminal older studies were retained when methodologically informative. Conference abstracts, non-English papers, editorials, commentaries, and reports lacking methodological detail were excluded.

Titles and abstracts were independently screened for relevance, and full-text articles were subsequently reviewed for eligibility. Extracted data included study aims, biomedical domain, data modality, AI technique, dataset characteristics, validation approach, performance metrics, and noted limitations. Extracted information also included dataset origin and type (public, institutional, or proprietary), sample size, and validation strategy (like k-fold cross-validation, hold-out, or external validation). However, many primary studies lacked complete reporting of these parameters, which was recorded as a limitation in the qualitative synthesis. The synthesis was performed narratively to characterize methodological trends, key clinical applications, and translational barriers; no quantitative meta-analysis or formal risk-of-bias assessment was undertaken given the heterogeneity of study designs.

**Figure 2:**
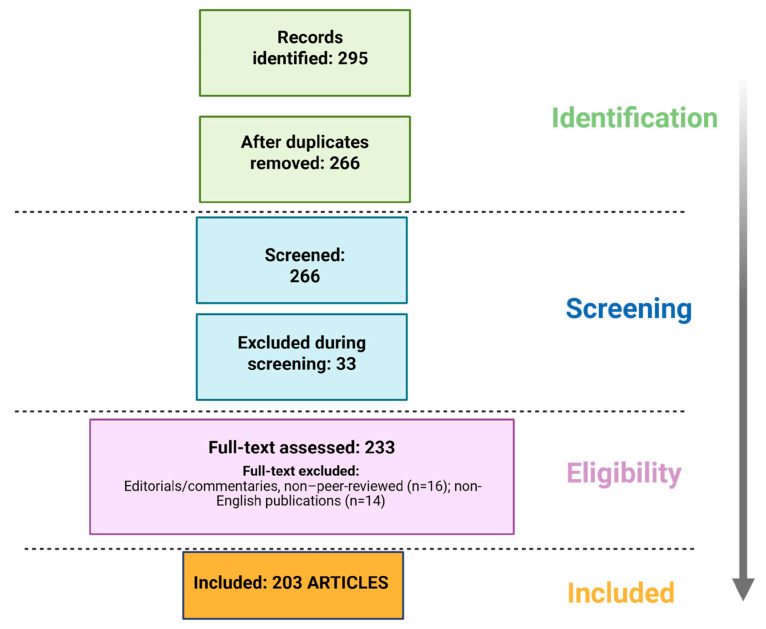
PRISMA Flow Diagram of Study Selection Process.

The literature selection process adhered to the PRISMA 2020 guidelines to ensure transparency and reproducibility. A total of 295 records were initially identified. After removing 29 duplicates, 266 unique studies were screened by title and abstract. Thirty-three records were excluded for lack of relevance, and 233 full-text articles were assessed for eligibility. Following exclusion of non–peer-reviewed materials (n = 16) and non-English publications (n = 14), a total of 203 articles were included in the final qualitative synthesis (Figure 2). This systematic review was conducted in accordance with the PRISMA 2020 Statement. The PRISMA 2020 flow diagram (Figure 1) is included in the main text, and the completed PRISMA 2020 checklist is provided as Supplementary Materials. The study has been registered on the Open Science Framework (OSF) to enhance transparency and reproducibility. The full protocol and metadata are available at https://osf.io/m4fq2 (accessed on 10 November 2025).

The methodological quality and potential risk of bias of the included studies were qualitatively appraised using domains adapted from the Joanna Briggs Institute (JBI) and AMSTAR 2 tools. Because of the substantial heterogeneity in study designs and reporting standards, a formal scoring system was not applied. Instead, the evaluation focused on the clarity of study objectives, validity of data sources, AI model validation methods, transparency of outcome reporting, and reproducibility of analyses. In numerous studies, performance metrics such as accuracy, sensitivity, specificity, or AUC were reported without sufficient contextual details, including dataset identifiers, validation folds, or training sample sizes. This lack of standardized reporting limits direct comparison across studies and constrains reproducibility, despite generally sound methodological design.

## 3. AI in Medicine

Table 1 provides a structured overview of the main AI applications discussed in this review across the four biomedical domains. It summarizes the types of data, computational tasks, and methodological approaches reported in the selected literature, offering a concise reference point that complements and anchors the narrative in this section.

### 3.1. AI in Cancer Therapy

In healthcare, AI systems function through specialized computational mechanisms that enable data interpretation and predictive analysis. ML algorithms, encompassing supervised, unsupervised, and reinforcement learning, detect patterns and relationships within medical datasets. In supervised learning, labeled data are used to train models such as Support Vector Machines (SVMs) or Random Forests (RF) to recognize abnormalities, including tumor regions in medical imaging [34]. Conversely, unsupervised learning methods discover hidden structures within unlabeled datasets, allowing the identification of distinct cancer subtypes, while reinforcement learning enhances therapeutic strategies by iteratively learning from previous patient outcomes [35]. Neural networks (NNs) emulate the brain’s information-processing structure through interconnected computational units known as nodes, which are organized into multiple layers. Input data pass through these layers and are transformed using activation functions like Rectified Linear Unit (ReLU) or Sigmoid, while optimization techniques such as backpropagation and gradient descent iteratively reduce prediction errors by adjusting connection weights [36].

Deep learning (DL), a branch of neural network–based AI, incorporates multiple hidden layers that enable the system to perform advanced biomedical analyses, including tumor segmentation and genomic data interpretation. Within DL, Convolutional Neural Networks (CNNs) excel at processing medical images by extracting spatial and contextual features, whereas Recurrent Neural Networks (RNNs) and Long Short-Term Memory (LSTM) architectures are suited for analyzing sequential or time-dependent patient information [37].

More recently, transformer-based models such as Bidirectional Encoder Representations from Transformers (BERT) and Generative Pre-trained Transformers (GPT) have been employed to analyze clinical narratives and biomedical literature using self-attention mechanisms, which allow models to focus on relevant portions of text for improved interpretation. To ensure reliability and interpretability, Explainable AI (XAI) frameworks are increasingly integrated into healthcare systems. These include SHAP (SHapley Additive exPlanations) and LIME (Local Interpretable Model-Agnostic Explanations), which quantify the influence of specific input variables on model predictions. Visualization methods, including heatmaps, further enhance interpretability by identifying areas of diagnostic importance in medical imagery [38,39,40].

#### 3.1.1. Emerging Methodological Developments

Recent advances in computational methods have further expanded the clinical applicability of AI in oncology. The following subsections outline key methodological innovations that enhance model performance, interpretability, and data privacy within cancer-related research and practice. Beyond conventional architectures such as CNNs, ResNets, and DenseNets, several methodological advances are increasingly shaping biomedical AI. Transfer learning enables pretrained models, originally developed for large-scale image datasets such as ImageNet, to be fine-tuned for specialized medical tasks with limited annotated data, substantially reducing training time and improving performance. Model interpretability frameworks, including Gradient-weighted Class Activation Mapping (Grad-CAM), SHAP and LIME, enhance transparency by visualizing or quantifying how input features influence model outputs, thus fostering clinical trust. In parallel, uncertainty quantification techniques, such as Bayesian neural networks and Monte Carlo dropout, are being adopted to estimate confidence in model predictions—an essential consideration for diagnostic reliability and clinical risk management. Together, these developments address major translational barriers in applying deep learning to real-world medical practice [41,42].

#### 3.1.2. Recent Paradigms in Biomedical AI

In addition to methodological refinements, several paradigm shifts are redefining how AI is deployed across biomedical oncology, enabling more collaborative, multimodal, and generalizable approaches to cancer prediction and management. In parallel with the development of traditional deep learning architectures, several new paradigms have emerged that redefine AI applications in biomedicine. Federated learning enables collaborative model training across multiple healthcare institutions without sharing sensitive patient data, thereby preserving privacy while expanding data diversity and model robustness. Multimodal fusion transformers integrate heterogeneous data sources, such as imaging, genomics, electronic health records, and clinical text, to provide holistic patient-level insights and improve predictive accuracy. Foundation models (like BioGPT, Med-PaLM, and CLIP-based architectures) represent a transformative approach in which large-scale pretrained models are fine-tuned for domain-specific medical tasks, offering unprecedented generalization and adaptability [43,44]. Collectively, these advances illustrate the rapid evolution of biomedical AI toward scalable, interpretable, and privacy-aware intelligent systems aligned with current (2024–2025) research directions.

Through these computational techniques, AI contributes significantly to cancer diagnosis, treatment response prediction, and personalized medical care, establishing itself as a cornerstone of modern healthcare innovation.

#### 3.1.3. Application of AI in Nanomedicine for Cancer Healthcare

AI has become a transformative force in the field of nanomedicine, profoundly influencing the ways in which cancer is diagnosed, treated, and monitored. Through the analysis of extensive datasets, AI-driven algorithms enhance the design and development of nanocarriers, predict cancer dynamics, and enable the creation of highly personalized therapeutic strategies. The integration of AI with nanotechnology represents a major step toward precision oncology, offering more targeted and effective treatment modalities.

In the context of nanocarrier design and drug delivery, AI methodologies such as ML and deep learning (DL) play a crucial role in the rational engineering of nanoparticles. These computational approaches can forecast optimal physicochemical properties, including particle size, morphology, surface charge, and functionalization, to achieve maximum therapeutic efficiency with minimal toxicity. Moreover, AI assists in optimizing drug-loading capacity and release kinetics, allowing nanocarriers to be adapted to the specific characteristics of individual tumor microenvironments [45].

Recent interdisciplinary studies illustrate how AI techniques directly enhance nanocarrier design and clinical translation. For example, CNNs have been used to predict the biodistribution and tumor uptake of gold nanoparticles and silica nanocarriers from PET/CT and fluorescence imaging data, facilitating precise dose optimization and minimizing off-target accumulation [46]. Deep learning frameworks have optimized liposomal nanoparticles for targeted chemotherapeutic delivery in breast and ovarian cancer, improving therapeutic indices and reducing systemic toxicity. In addition, AI-assisted radiomics combined with MRI and photoacoustic imaging enables the detection of iron oxide-based nanoparticles, providing non-invasive tracking of drug release and treatment response. Reinforcement learning models have also guided the adaptive control of polymeric and lipid nanocarriers for on-demand drug release, demonstrating early translational feasibility in preclinical oncology. Collectively, these examples show how AI connects nanoscale engineering with clinically actionable theranostic strategies [47].

AI-based predictive modeling also contributes substantially to cancer targeting. By simulating nanoparticle interactions within biological systems, these models can estimate biodistribution, tumor localization, and potential off-target effects with high precision. In silico simulations further support the identification of suitable biomarkers and ligands for nanoparticle surface modification, leading to the development of advanced delivery systems that improve therapeutic success while minimizing systemic side effects [48,49].

Another major application lies in the personalization of cancer therapy. AI algorithms integrate genomic, proteomic, and clinical data to generate patient-specific treatment regimens. By forecasting individual responses to nanomedicine-based therapies, AI enables the selection of the most effective nanoparticle formulations and drug combinations. This personalized approach is particularly valuable in addressing tumor heterogeneity and reducing the likelihood of treatment resistance [50].

The use of AI in real-time monitoring has also advanced cancer management. When coupled with nanoparticle-based contrast agents, AI-enhanced imaging systems provide continuous insights into tumor development and therapeutic response. These algorithms dynamically analyze imaging outputs, allowing clinicians to adjust treatment strategies promptly to improve efficacy. Additionally, the combination of AI with wearable biosensors and nanoscale monitoring devices facilitates continuous patient observation, supporting the early identification of complications and timely intervention [51].

Finally, AI plays a pivotal role in accelerating the drug discovery process. By analyzing vast chemical libraries and predicting interactions between nanoparticles and biological targets, AI models can identify the most promising drug–nanocarrier combinations. This capability streamlines formulation development and significantly reduces both the cost and duration associated with bringing novel therapies to clinical application [52].

The convergence of AI with nanotechnology is driving innovative integrations that further enhance cancer healthcare:

(a) Theranostic nanoplatforms: AI-powered theranostic platforms combine diagnostic and therapeutic functions within a single nanostructure. These platforms can detect cancer biomarkers, deliver targeted therapy, and monitor treatment responses in real-time, offering a comprehensive personalized management solution [53].
(b) AI-driven nano-robotics: AI-controlled nano-robots demonstrate promise in precise drug delivery and tumor targeting. These nano-robots autonomously navigate through the bloodstream, identify cancer cells, and release therapeutics in a controlled manner, minimizing damage to healthy tissues [54].
(c) Multi-omics data integration: AI algorithms integrate genomic, transcriptomic, and proteomic data with nanomedicine approaches to uncover novel biomarkers and predict therapeutic responses. This integration enhances patient stratification and informs the development of personalized nano-therapeutics [55].
(d) Quantum computing for nanomedicine: Quantum computing, combined with AI, enables rapid simulation of complex biological environments. This enhances nanoparticle modeling and expedites the development of next-generation nanomedicines [56].

### 3.2. AI in Cardiology

The integration of artificial intelligence (AI) into patient monitoring systems offers numerous advantages in improving healthcare outcomes. Through AI-based algorithms, vital signs such as heart rate, blood pressure, and respiratory rate can be continuously monitored in real time, enabling early detection of abnormalities and timely medical interventions. This proactive approach enhances patient safety and treatment efficiency. Hannun et al. demonstrated that AI algorithms are capable of detecting arrhythmias and ischemic changes from electrocardiogram (ECG) data, facilitating the early diagnosis of conditions such as atrial fibrillation [57]. The advancement of AI-driven monitoring devices has further enabled real-time feedback and automated analysis of physiological parameters across various clinical settings [58]. Recent research has introduced innovative methods to enhance ECG interpretation, including the application of two event-related moving averages in combination with the fractional Fourier transform (FrFT), significantly improving peak detection accuracy and cardiac condition classification [59]. These technologies hold great promise in cardiology, particularly for the continuous detection of critical events such as malignant arrhythmias [60]. Moreover, by rapidly processing vast datasets with high precision, AI systems can reduce the workload of healthcare professionals while improving diagnostic accuracy and overall patient management [61].

#### 3.2.1. AI in Heart Failure (HF)

Most traditional ECG indices based on voltage exhibit relatively low sensitivity, typically ranging from 19% to 25%. The incorporation of AI into ECG analysis has been shown to substantially improve sensitivity for detecting left ventricular hypertrophy (LVH), increasing it from 42% to 69%, though this improvement is accompanied by a modest decrease in specificity from 92–94% to 87% [62]. This trade-off suggests that while AI enhances early disease detection, it also increases the likelihood of false-positive results, which may necessitate additional follow-up evaluations. In clinical practice, especially in real-world settings, careful management of this balance is critical to avoid overburdening healthcare systems while maximizing the benefit of early detection.

Despite these advancements, AI-based ECG analysis raises certain clinical concerns. Enhanced sensitivity allows AI models to identify subtle or early abnormalities that conventional methods may overlook; however, reduced specificity can lead to overdiagnosis and unnecessary interventions. In medical decision-making, particularly in populations such as infants, the trade-off between sensitivity and specificity must be carefully aligned with disease prevalence and the risks associated with false positives and false negatives. The application of AI for predictive modeling has also introduced novel risk factors, such as endothelin-1, which is associated with oxidative stress and cardiac remodeling [63]. Nevertheless, further research, including randomized trials, is required to validate the effectiveness of AI-guided therapeutic strategies and to translate predictive findings into meaningful improvements in patient care.

Meta-analyses on AI-enhanced ECG detection indicate that AI can elevate sensitivity from the 19–25% range typical of traditional methods up to 69%, while slightly reducing specificity from 92–94% to 87%, thereby improving overall diagnostic accuracy for LVH [62]. Several machine learning models have been proposed to advance clinical diagnosis. For instance, one study utilized a CNN trained on 12-lead ECG data to diagnose left ventricular diastolic dysfunction (LVDD) in patients presenting with dyspnea, outperforming NT-proBNP in distinguishing cardiac from pulmonary causes. Another multicenter prospective study employed ML to evaluate LVDD using ECG in comparison to echocardiographic measures of left ventricular relaxation velocities (e′), demonstrating strong correlations with sensitivity of 78%, specificity of 77%, negative predictive value of 73%, and positive predictive value of 82% in internal testing, with similar performance in external validation [64].

Beyond ECG, AI has also been applied to other imaging modalities for the assessment of structural heart disease. Techniques such as radiomics, which extract quantitative imaging features through algorithmic analysis, have demonstrated improved diagnostic capabilities. For example, AI-driven radiomic analysis correctly differentiated hypertrophic cardiomyopathy (HCM) from hypertensive heart disease with an accuracy of 85.5%, significantly outperforming traditional methods, which achieve approximately 64% accuracy [65,66].

#### 3.2.2. AI in Coronary Artery Disease

Among the various AI applications in cardiology, systems for Fractional Flow Reserve using CT (FFR-CT) have demonstrated particularly promising diagnostic performance in the assessment of coronary artery disease (CAD). In a comparative study, Lipkin et al. evaluated coronary computed tomography angiography (CCTA) interpreted with AI quantitative CT (AI-QCT) against myocardial perfusion imaging (MPI) for detecting obstructive CAD. AI-QCT achieved a higher area under the curve (AUC) than MPI (0.88 vs. 0.66) for predicting stenosis greater than 50% in 301 patients enrolled in the CREDENCE trial [67]. Similarly, Chiou et al. compared AI-QCTISCHEMIA with CT-FFR and physician visual interpretation in 442 patients, reporting superior specificity and diagnostic performance for AI-QCTISCHEMIA relative to both clinician interpretation (specificity 0.62) and CT-FFR (specificity 0.76) [68].

Despite these promising results, several challenges remain. The computational demands of AI algorithms and the need for large, well-annotated datasets are significant limiting factors. Pershina et al. highlighted that the diagnostic accuracy of FFR-CT (AUC = 0.90) is highly dependent on the quality of the input data and available computational resources [69]. Additionally, population homogeneity in many studies introduces bias, as AI-based CT-FFR analyses frequently exclude high-risk patients, limiting generalizability to broader clinical populations [70]. Another important consideration is the trade-off between accuracy and interpretability. While models such as DenseNet201 achieve high accuracy, their “black-box” nature can impede clinical trust and slow adoption. DenseNet121 outperformed thoracic radiologists in certain tasks; however, the lack of transparency in these models remains a barrier to routine clinical integration [71].

Several deep learning architectures, including EfficientNet-B0, DenseNet201, ResNet101, Xception, and MobileNet-v2, have been proposed for automated coronary artery segmentation and classification. Among these, DenseNet201 showed the strongest performance, achieving an accuracy of 0.90, specificity of 0.9833, positive predictive value (PPV) of 0.9556, Cohen’s Kappa of 0.7746, and an AUC of 0.9694, underscoring its superiority in classification tasks [72]. Beyond imaging, AI has also been applied to analyze biochemical markers for cardiovascular risk assessment. For example, Xue et al. employed unsupervised machine learning to stratify ST-segment elevation myocardial infarction (STEMI) patients into distinct phenogroups based on lipid profiles. Statistical analyses, including ANOVA and Cox proportional hazards models, demonstrated significant differences among the phenogroups in lipoprotein(a), high-density lipoprotein cholesterol (HDL-C), and apolipoprotein A1 (ApoA1) patterns, providing predictive insights for clinical outcomes [73].

#### 3.2.3. CT vs. MRI

As shown in Table 2, AI integration diverges substantially between CT and MRI, reflecting modality-specific characteristics in data structure, computational preprocessing, and downstream clinical interpretability within cardiovascular diagnostics.

### 3.3. AI in Neuronal Diseases

#### 3.3.1. Evolution of AI in Neurological Diagnostics

Early applications of artificial intelligence in neurology primarily relied on conventional machine learning algorithms that used manually selected features extracted from structured datasets, neuropsychological evaluations, and basic neuroimaging results. Developing these systems often required substantial domain expertise to pinpoint informative predictors, and their performance was frequently constrained by limited scalability and poor generalization to heterogeneous patient cohorts. Nevertheless, these early models were instrumental in proving that automated decision-support systems could be effectively applied to neurological contexts, paving the way for more adaptive and data-driven learning approaches.

The emergence of deep learning marked a significant transformation in this area, enabling models to process unstructured and high-dimensional data directly, such as MRI images, EEG recordings, vocal characteristics, and movement sensor outputs [95,96]. Deep architectures including CNNs and RNNs greatly enhanced the ability to identify patterns and extract complex features, yielding more precise and sophisticated diagnostic predictions without depending on manually designed input variables. This technological leap has made it possible to uncover subtle biomarkers linked to neurological diseases, such as Alzheimer’s disease, Parkinson’s disease, and multiple sclerosis, at earlier stages and with higher diagnostic accuracy.

Furthermore, incorporating multiple data modalities into unified deep learning frameworks offers a more comprehensive perspective on a patient’s neurological status. This multimodal integration supports the ongoing transition from traditional symptom-focused evaluation to precision neurology grounded in data analytics. Collectively, these developments mark an essential advancement toward scalable, AI-driven diagnostic solutions capable of reshaping both individual patient care and large-scale neurological screening initiatives.

#### 3.3.2. AI in Parkinson Disease (PD)

Among the numerous domains explored for artificial intelligence applications in Parkinson’s disease (PD), neuroimaging remains one of the most comprehensively investigated [97,98]. The combination of dopamine transporter (DaTscan) imaging with CNNs has achieved exceptional performance in differentiating PD patients from healthy individuals [99]. Recent investigations have reported classification accuracies surpassing 95% through deep learning-based interpretation of DaTscan images—significantly improving upon the results obtained via conventional visual assessment [100,101].

Applications of structural and functional MRI have also yielded encouraging outcomes, both for early diagnosis and for tracking disease progression [102,103]. In particular, graph neural networks (GNNs), a class of deep models designed to process data structured as graphs, where brain regions are treated as nodes and their functional interactions as edges, have been successfully applied to resting-state functional connectivity analyses. These approaches have achieved classification accuracies ranging from 88% to 92% when distinguishing PD cohorts from control groups [104]. By capturing intricate topological patterns and network-level relationships within the brain, GNNs provide new insights into the neural connectivity alterations characteristic of PD and other neurological disorders.

Furthermore, diffusion tensor imaging (DTI) analyzed with advanced machine learning frameworks has uncovered subtle microstructural abnormalities in white matter tracts that may emerge even before the onset of clinical manifestations [105,106]. These findings underscore the potential of AI-enhanced neuroimaging to facilitate earlier and more precise detection of PD-related pathophysiological changes.

Voice alterations are recognized as some of the earliest non-motor indicators of Parkinson’s disease (PD), often manifesting several years prior to the appearance of clinically measurable motor symptoms [107,108]. These vocal abnormalities, characterized by reduced volume, monotonous tone, breathiness, and subtle articulation deficits, can be easily missed during standard neurological evaluations. Nonetheless, they offer a promising window for early identification of PD, particularly in cases where conventional diagnostic approaches may not yet reveal overt signs of pathology.

The incorporation of AI into voice analysis has considerably improved the accuracy and reliability of detecting vocal biomarkers linked to PD. Through the extraction of acoustic parameters such as fundamental frequency variability, jitter, shimmer, harmonics-to-noise ratio, and various spectral attributes, AI-based models have achieved diagnostic accuracies ranging between 85% and 93% [109,110]. These findings highlight the potential of voice analysis as a non-invasive and scalable screening approach, particularly suited for remote assessment and early-stage community-level detection efforts.

Building upon these foundations, recent work has leveraged deep learning frameworks to move beyond traditional signal-processing pipelines. RNNs, and notably LSTM architectures, have proven highly effective in capturing temporal dependencies and sequential variations embedded within speech patterns, thereby modeling the dynamic progression of PD-related vocal changes [111]. More recently, transformer-based architectures, initially developed for natural language processing, have demonstrated substantial promise in learning long-range contextual relationships in speech sequences. By enabling models to train directly on raw or minimally preprocessed audio signals, these approaches minimize the reliance on hand-engineered features and facilitate end-to-end disease classification.

Ultimately, AI-driven voice analysis provides a cost-efficient, non-invasive, and highly scalable diagnostic pathway, offering opportunities for continuous disease monitoring, real-time clinical feedback, and broad integration into telehealth and digital health ecosystems [112].

Gait impairments are among the most distinctive and diagnostically significant motor manifestations of Parkinson’s disease (PD), typically presenting as short, shuffling steps, diminished arm swing, postural instability, and freezing of gait episodes. These changes in locomotion serve as objective and quantifiable indicators for both disease onset and progression. In recent years, AI has been increasingly utilized to analyze these movement abnormalities through data derived from wearable motion sensors, such as accelerometers and gyroscopes. When positioned on various body parts, including the feet, waist, or limbs, these sensors capture high-resolution motion data during walking tasks.

By training machine learning algorithms on such data, researchers have achieved highly accurate classification of PD patients, uncovering intricate gait-related patterns that often elude conventional clinical observation. In some investigations, the sensitivity and specificity of AI-based gait analysis for early PD detection have surpassed 90%, even in scenarios where traditional clinical evaluations provide ambiguous results [113,114]. This level of precision has established gait analysis as a powerful diagnostic and monitoring tool, facilitating both early identification and longitudinal tracking of motor dysfunction in PD.

Beyond wearable technologies, the emergence of AI-driven computer vision techniques has expanded gait analysis into non-contact and highly scalable modalities. Modern markerless motion capture systems can now assess walking behavior using ordinary video recordings obtained from smartphones or surveillance cameras. By extracting joint trajectories and body kinematics from these recordings, deep learning models can detect gait irregularities that signal PD-related motor decline. This approach provides a low-cost and accessible alternative to specialized hardware, enabling movement assessment in diverse environments, including homes, clinics, and public spaces (Table 3) [115].

Furthermore, the integration of such AI systems within telemedicine platforms allows for continuous, remote evaluation of motor symptoms, an especially valuable feature for patients in underserved or rural areas with limited access to movement disorder specialists [116]. As AI methodologies continue to advance, they are poised to redefine clinical and research approaches to gait assessment in Parkinson’s disease, enhancing both precision diagnostics and personalized disease management (Table 4).

**Table 3:** Assessment of the digital biomarkers and smartphone applications.

Aspect	Description	Ref.
Role of Smartphone Technology in PD Assessment	The widespread availability and computing power of smartphones have enabled the development of accessible, non-invasive, and scalable digital biomarker platforms for PD monitoring. These systems use built-in sensors and software to capture behavioral and physiological data.	[117,118]
Motor Assessment through Sensor-Based Applications	Finger-tapping apps measure motor speed and variability, serving as indicators of bradykinesia.	[119,120]
Speech-Based Biomarkers	Voice recording applications analyze speech fluency and tremor-related vocal disruptions, key symptoms of PD.	[119,120]
Remote Monitoring and Telehealth	Smartphone-based biomarkers allow continuous and passive monitoring of patients in real-world environments. This enhances personalized care, supports timely interventions, and improves patient engagement.	[121,122]
Application in Low-Resource Settings	These technologies provide cost-effective screening and early detection solutions for rural or resource-limited areas, helping reduce healthcare disparities.	[121,122]
Advanced Computational Capabilities	Modern smartphones perform real-time signal processing using edge computing and machine learning to analyze tremor patterns, gait variability, and speech signals directly on the device, ensuring privacy and faster feedback.	[123]
Federated Learning Approaches	Enable continuous improvement of diagnostic algorithms without sharing sensitive data, enhancing personalization and accuracy across diverse populations.	[123]

**Table 4:** AI in Parkinson’s Disease (PD) Treatment Optimization and Personalized Medicine.

Aspect	Description	Ref.
AI in Treatment Optimization	AI and machine learning are transforming PD treatment by analyzing complex patient response patterns to dopaminergic therapy. These models integrate longitudinal data such as symptom fluctuations, medication adherence, and side-effect profiles to predict individual treatment efficacy more accurately than traditional methods.	[124,125]
Personalized Pharmacological Regimens	Predictive modeling allows clinicians to tailor drug dosages and schedules to individual patients, minimizing adverse drug reactions and enhancing therapeutic outcomes.	[124,125]
AI-Driven Decision Support Systems	Integrated into electronic health records, these systems assist clinicians with real-time dosage adjustments and dynamic care models.	[126,127]
Deep Reinforcement Learning in Neuromodulation	AI algorithms fine-tune deep brain stimulation (DBS) by simulating different stimulation scenarios and learning from patient feedback to optimize therapeutic outcomes and minimize side effects.	[128,129]
Improved Clinical Efficiency and Quality of Life	Intelligent DBS optimization reduces clinician workload, resource use, and patient side effects, improving quality of life and clinical efficiency.	[128,129]
Predictive Modelling for Disease Progression	Advanced ML models combine clinical, imaging, genetic, and digital biomarker data to predict long-term outcomes such as motor complications, cognitive decline, and quality of life deterioration.	[130]
Risk Stratification and Patient Selection	AI tools identify patients most likely to benefit from interventions like DBS or clinical trials, supporting precision medicine and resource optimization.	[130]

Despite the substantial progress made in applying artificial intelligence (AI) to Parkinson’s disease (PD) diagnostics, several critical challenges continue to hinder its clinical translation. Among the most prominent issues is data heterogeneity. Current research efforts frequently employ diverse methodologies, imaging techniques, sensor configurations, and clinical assessment tools, leading to inconsistencies across datasets. Such variability complicates data harmonization and limits model generalizability, as algorithms trained on one dataset often underperform when tested on another. Additionally, many AI models are developed using small-scale or demographically narrow cohorts, which increases the risk of algorithmic bias and reduces performance when applied to broader and more diverse populations [131,132]. Insufficient representation across age groups, ethnic backgrounds, and disease phenotypes raises concerns about fairness, reliability, and clinical applicability in real-world diagnostic contexts [133,134].

The widespread adoption of smartphones and wearable sensors for continuous PD monitoring also introduces security and privacy risks that must be addressed. Studies have shown that motion sensors embedded in smartphones can be manipulated for keystroke inference attacks, potentially compromising user privacy during data entry. Similarly, wireless sensor networks used in gait or tremor tracking are vulnerable to physical layer fingerprinting attacks, enabling malicious actors to bypass authentication and gain access to sensitive health information. These vulnerabilities are particularly concerning in long-term monitoring systems, where data related to patients’ motor function is transmitted frequently. Therefore, effective implementation frameworks must include strong encryption mechanisms, secure communication protocols, and privacy-preserving analytic methods to ensure that patient confidentiality is protected without compromising the clinical utility of AI-based tools.

Beyond technical and ethical considerations, regulatory and operational barriers further complicate the path to clinical integration. The approval pathways for AI-driven medical technologies remain in flux, as organizations such as the FDA and EMA continue adapting traditional regulatory structures to accommodate adaptive and continuously learning systems. This evolving landscape often results in delays in authorization and deployment, restricting the timely application of innovative solutions in patient care [135]. Moreover, incorporating AI systems into existing clinical workflows requires significant organizational adaptation. Healthcare professionals need to understand, interpret, and trust AI-generated insights, and the user interfaces of these systems must be intuitive and supportive rather than disruptive to established decision-making processes. Achieving interoperability with electronic health records (EHRs) and ensuring that AI outputs align with clinical pathways are equally crucial for effective implementation and user adoption [136,137].

Overall, these multidimensional challenges highlight the necessity for collaborative, interdisciplinary efforts involving clinicians, data scientists, ethicists, and regulatory authorities to fully realize the potential of AI-enhanced diagnostics and management in Parkinson’s disease.

#### 3.3.3. AI in Alzheimer’s Disease (AD)

After image preprocessing and segmentation, scans are ready for computational interpretation. AI methods have streamlined Alzheimer’s research by improving diagnostic workflows and classification, though machine-learning results vary in robustness and reproducibility. Deep learning, multilayer neural networks capable of extracting complex patterns, has been especially useful for detecting MRI atrophy, PET biomarkers, and combined PET–MRI/fMRI signatures. Below, we review the most common AI architectures used to distinguish Alzheimer’s disease, mild cognitive impairment, and healthy controls; the principal imaging modalities include structural/functional MRI, PET tracers, and PET/MRI fusion (Table 5).

#### 3.3.4. Deep Learning in AD Diagnosis and Classification

Among deep learning techniques, the most frequently cited models across reviewed studies include CNNs, RNNs, autoencoders, and generative adversarial networks (GANs). The majority of research efforts have focused on AD diagnosis and classification, with CNN-based architectures emerging as the dominant approach. CNNs process structured input data—such as medical imaging—through a hierarchy of interconnected layers (input, hidden, and output). These networks apply convolutional filters, small sliding matrices designed to capture spatial features such as edges, textures, and contours, which are subsequently used for feature extraction and classification [152]. Numerous investigations have demonstrated the strong performance of CNNs in multimodal imaging-based AD detection, achieving high classification accuracy. The Visual Geometry Group Network (VGGNet) represents one of the earliest and most widely implemented deep CNN architectures in AD research [153,154,155]. Its design minimizes error rates by employing fewer kernel features while increasing the depth of the network [156]. In a recent study, Kim et al. [153] developed a highly accurate hybrid model that integrated VGGNet with a one-dimensional CNN designed to capture brain contour information, specifically focusing on cortical and subcortical boundaries and shape configurations. The incorporation of VGGNet improved model precision significantly, achieving 0.986 accuracy, and outperformed standard versions such as VGG-16, VGG-19, and AlexNet. Optimal performance was observed with an input size of 256 × 256 [153]. Similarly, Mujahid et al. proposed an ensemble framework combining VGG-16 and EfficientNet-B2, which enhanced early AD detection accuracy [154].

Another prominent CNN architecture, ResNet, introduces residual connections that enable efficient information propagation through multiple layers, reducing computational overhead while preventing gradient degradation [157]. Various ResNet variants have been employed in AD classification and early disease identification [158,159,160,161,162,163,164]. For example, Odusami et al. utilized ResNet18 to classify functional MRI (fMRI) data, reporting 99.99% accuracy in differentiating early mild cognitive impairment (MCI) from AD [158].

The DenseNet architecture enhances feature reuse by connecting each layer to every other layer through dense feed-forward connections, ensuring efficient information flow and minimizing redundancy [156]. DenseNet has been effectively implemented for automated feature extraction and AD diagnosis [165]. In a comparative evaluation, Carcagnì et al. analyzed several CNN architectures—including DenseNet, ResNet, and EfficientNet, and found that deeper versions of DenseNet and ResNet outperformed shallower models such as VGG, yielding a 7% improvement in MRI-based AD detection accuracy [166]. Similarly, Sharma et al. [167] introduced a hybrid AI model that combined transfer learning with DenseNet-121 and DenseNet-201, integrated with machine learning classifiers, achieving 91.75% accuracy and 96.5% specificity.

Beyond these major CNN families, several other architectures have contributed to AD diagnosis and classification [168,169,170,171,172,173,174,175]. For instance, the Dementia Network (DemNet) achieved 95.23% accuracy and an AUC of 0.97 for AD staging using non-MRI data [176], while AlzheimerNet demonstrated superior classification precision over traditional approaches. The LeNet architecture, one of the earliest CNN designs, utilizes MaxPooling layers to reduce feature map dimensionality by discarding low-importance data [177]. A modified LeNet model proposed by Hazarika et al. attained a classification accuracy of 96.64% in AD identification [178].

In contrast to CNNs, RNNs are optimized for capturing temporal dependencies within sequential data, allowing effective modeling of time-dependent variations [177]. Mahim et al. integrated gated RNNs with a vision transformer (ViT) architecture, leveraging the ability of gated networks to utilize contextual information from previously processed data. This hybrid RNN–ViT model achieved 99.69% accuracy for binary classification of MRI scans in AD detection [178,179].

Autoencoders, by comparison, function as unsupervised learning mechanisms that compress input data into latent representations and subsequently reconstruct the original input while preserving essential features [180]. Al-Otaibi et al. introduced a dual-attention convolutional autoencoder, demonstrating 99.02% real-time accuracy in AD recognition based on MRI data [181]. Another study employing fMRI developed a specialized autoencoder to effectively differentiate normal aging from AD progression, also reporting excellent classification results [182].

Finally, GANs have emerged as powerful tools in medical image synthesis and domain adaptation, comprising two competing neural networks, one generating synthetic images and the other evaluating them [183]. In 2023, a Loop-Based GAN for Brain Network (BNLoop-GAN) was introduced to model the distribution of brain connectivity networks using multimodal imaging data. The approach successfully discriminated between healthy controls and AD patients with 81.8% sensitivity and 84.9% specificity, outperforming other models across resting-state fMRI and structural MRI modalities [184,185]. Similarly, Chui et al. [186] employed a GAN framework integrated with CNNs and transfer learning to augment underrepresented data, improving the accuracy and robustness of AD classification across multiple datasets.

#### 3.3.5. Prediction/Prognosis

AD research has expanded beyond diagnosis, increasingly focusing on early detection and prognosis. Recent models integrate MRI, PET, molecular, and clinical data to identify biomarkers that accurately track disease progression. Longitudinal analyses have enabled the monitoring of volumetric and metabolic brain changes using AI-driven statistical techniques, such as linear mixed-effects models [187]. The fusion of imaging data with cognitive measures provides a more comprehensive understanding of AD development.

Predictive modeling has gained momentum due to its potential for early intervention. Aqeel et al. applied an RNN with LSTM to predict neuropsychological and MRI biomarkers, distinguishing AD from MCI [188]. Khalid et al. achieved 99.7% accuracy and an AUC of 0.99 using a feed-forward network combining GoogLeNet and DenseNet-121 [189]. Similarly, deep learning tools applied to MRI datasets have shown over 80% accuracy in dementia staging [190].

Several models specifically target MCI-to-AD conversion. Peng et al. used PET-based radiomics and clinical scales (CDR, ADAS) with multivariate logistic regression, achieving 87% sensitivity and 78% specificity [191]. Lin et al. utilized an extreme learning machine (ELM) across five imaging modalities, demonstrating high predictive accuracy [192]. Fakoya et al. developed a CNN model combining MRI and PET slices, preserving modality-specific features while maintaining 94.0% accuracy [193].

AI tools have also explored structural and functional biomarkers. Pan et al. proposed an Ensemble 3DCNN to map MRI-based structural alterations [194], while Kim et al. 2021 applied autoencoders to predict disease progression across AD stages [195]. Brain age prediction models, such as the BrainAGE framework [196], further contributed to longitudinal AD studies.

Key pathological markers like amyloid-beta and tau remain central to AI-based predictions. Wang et al. employed tau-PET images with support vector regression to estimate brain age [197]. Chattopadhyay et al. applied deep learning to T1-weighted MRI for Aβ plaque prediction, showing strong potential in MCI prognosis [198]. Moreover, cognitive performance prediction using imaging data has gained attention. Habuza et al. developed a CNN regression model that differentiated normal and MCI subjects with an AUC of 99.57% [199], while Liang et al. used a multi-task learning framework to predict cognitive decline based on structural associations [200].

### 3.4. AI in Liver Diseases

Artificial intelligence is reshaping multiple facets of hepatology, from tissue analysis to surgery and transplant decision-making. In histopathology, AI tools are increasingly applied to digitized slides, a shift still limited by the incomplete adoption of whole-slide imaging (WSI) and non-standardized acquisition formats, but both retrospective and prospective multicenter studies remain feasible by rescanning and harmonizing stained paraffin blocks [201]. Digital methods help reduce well-known interobserver variability in pathology and radiology (previously demonstrated for liver cancer), supporting more reproducible diagnostic and prognostic workflows [202]. As a result, histopathology has become one of the fastest-growing AI application areas in hepatology after imaging, with algorithms now assisting clinicians in identifying disease-specific features and suggesting likely diagnoses and stratifications [203,204].

Robotic platforms and AI are also extending the capabilities of liver surgery and living-donor hepatectomy. Minimally invasive and robotic approaches have been shown non-inferior to open techniques and can improve donor safety, shorten hospitalization, and speed functional recovery for recipients and donors alike [205,206]. Robotic systems shorten the technical learning curve versus purely laparoscopic approaches and enable advanced teaching through dual consoles and virtual simulation environments, while integration of preoperative 2D/3D imaging, intraoperative ultrasound, and emerging AI guidance promises progressively greater autonomy and precision in the operating room [207].

In transplantation, AI offers an opportunity to move allocation and wait-list management toward precision medicine. Machine-learning models have outperformed conventional scoring in several exploratory studies by better capturing patient trajectories and complex predictors of wait-list mortality [208,209]. For example, ML-derived algorithms (e.g., OPOM) have shown improved short-term mortality prediction over MELD in retrospective registry analyses and simulation studies, suggesting potential reductions in wait-list deaths and changes in allocation equity. Similarly, hybrid ML–statistical approaches have been used to predict HCC dropout, producing competitive discrimination metrics in validation cohorts [210]. Nevertheless, these encouraging findings remain exploratory: current guidelines stress the need for careful external, prospective validation and simulation testing before clinical deployment to identify biases and ensure safe, equitable adoption.

Together, these developments illustrate a broad AI footprint across hepatology, from automated slide interpretation and imaging augmentation to intraoperative support and smarter transplant prioritization, while underscoring persistent challenges: data standardization, model interpretability, population diversity in training sets, and rigorous prospective validation.

## 4. Qualitative Appraisal of Study Quality

Overall, the included studies exhibited moderate methodological quality. Most clearly stated their objectives and used valid datasets; however, external validation of AI models was inconsistently reported. Approximately half of the studies provided detailed performance metrics such as accuracy, sensitivity, and AUC values, while others reported results narratively without standardized measures. Reporting transparency and data availability were often limited, which may increase risk of bias and reduce reproducibility. Only a small number of studies explicitly described reproducibility strategies or open-source code sharing. Despite these limitations, the majority demonstrated methodological soundness sufficient to support the synthesized conclusions.

### 4.1. Advantages, Limitations, and Mitigation Strategies of Artificial Intelligence in Biomedicine

**Figure 3:**
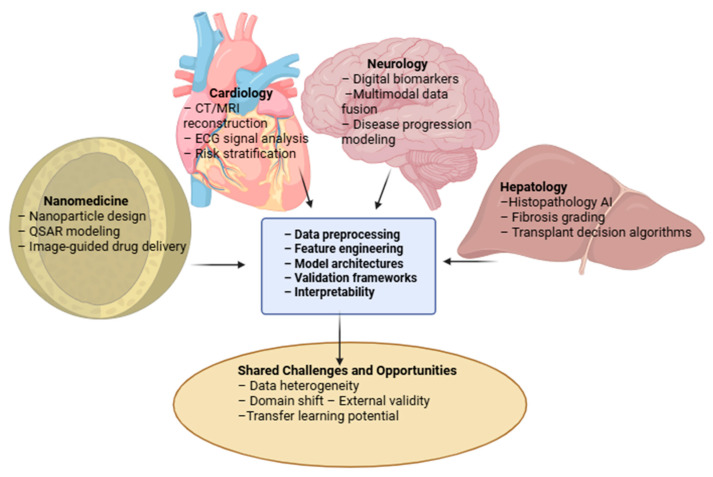
Cross-domain integration map illustrating AI applications in nanomedicine, cardiology, neurology, and hepatology. Domain-specific use cases converge on shared computational principles—data preprocessing, feature engineering, model architectures, validation frameworks, and interpretability—while the lower panel highlights overarching challenges and opportunities, including data heterogeneity, domain shift and external validity, and transfer learning potential.

The integration of AI technologies into biomedical research and clinical care offers substantial advantages but also introduces new challenges. While the domain-specific sections of this review discuss these aspects in detail, the following synthesis provides a consolidated perspective useful for clinicians, regulators, and researchers. The relationships among the four biomedical domains and their convergence toward shared AI workflows are summarized in Figure 3.

AI enables large-scale data analysis, high-dimensional pattern recognition, and automated image interpretation far beyond human capability. It accelerates diagnostic workflows, supports personalized medicine through predictive modeling, and enhances treatment planning via multimodal data integration. In research contexts, AI allows hypothesis generation from complex datasets, facilitating drug discovery and biomarker identification that were previously infeasible.

Despite its potential, AI implementation faces several challenges. Data bias and lack of representativeness may lead to skewed predictions, particularly across demographic or institutional boundaries. Model interpretability remains limited, complicating clinical validation and trust. Overfitting and false positives can arise when models are trained on small or unbalanced datasets. Furthermore, AI integration may impose technical burdens on existing clinical workflows, requiring new infrastructure, continuous updates, and clinician training.

To enhance reliability and clinical adoption, rigorous prospective multicentre validation is essential. Implementing explainability and interpretability metrics (like SHAP, LIME, Grad-CAM) can improve transparency. Data harmonization and standardized acquisition protocols reduce bias and improve generalizability. Continuous model monitoring and adaptive retraining ensure long-term performance stability, while regulatory frameworks and ethical oversight remain key to patient safety and accountability.

### 4.2. Regulatory and Governance Context of AI in Healthcare (2024–2025)

The global regulatory landscape for Artificial Intelligence (AI) in healthcare has evolved rapidly, particularly concerning adaptive and continuously learning models. Current frameworks emphasize safety, transparency, data governance, and ongoing performance monitoring, ensuring that AI-based medical tools maintain reliability throughout their life cycle.

United States (FDA). The U.S. Food and Drug Administration (FDA) finalized its Predetermined Change Control Plan (PCCP) guidance in late 2024 for software functions, including AI/ML components. The PCCP specifies how developers must define anticipated post-authorization modifications to algorithms, verification methods, and validation criteria, an essential step for regulating adaptive models [211]. This builds upon the Good Machine Learning Practice (GMLP) principles jointly published by the FDA, Health Canada, and the UK MHRA, which outline standards for data quality, separation of training and testing datasets, and documentation of model versioning [212].

European Union. The EU Artificial Intelligence Act (Regulation (EU) 2024/1689) entered into force on 1 August 2024, complementing the existing Medical Device Regulation (MDR) and In Vitro Diagnostic Regulation (IVDR) [213]. AI systems used in healthcare are generally classified as “high-risk,” requiring strict conformity assessments, risk management procedures, and post-market monitoring. The AI Act introduces explicit obligations for data governance, transparency, human oversight, and harmonized technical documentation, aiming to ensure both safety and ethical accountability across the EU.

United Kingdom (MHRA). The Software and AI as a Medical Device (SaMD/AIaMD) Change Programme continues to modernize the UKCA regulatory framework, aligning it with international GMLP principles. The program defines requirements for evidence generation, validation, and monitoring of AI systems following deployment, emphasizing clinical safety and explainability [214].

Global alignment. The International Medical Device Regulators Forum (IMDRF) provides the foundational definitions and risk-categorization framework for SaMD, widely adopted by the FDA, EU, and MHRA [215]. Recent ISO standards—including ISO/IEC 23894 (AI risk management) [216] and ISO/IEC 42001 (AI management systems) [217]—can be integrated into existing quality management systems (ISO 13485, ISO 14971, IEC 62304) [218,219,220]. Additionally, the World Health Organization (WHO) has published ethical and governance guidelines for large multimodal AI models, focusing on data provenance, auditability, and human oversight [221].

To support safe and reproducible deployment of AI in healthcare, several practical steps are recommended:

1. Proper classification as medical device software (SaMD) following IMDRF risk categories to determine the required level of clinical evidence [215].
2. Implementation of a Predetermined Change Control Plan (PCCP) early in development—clearly defining what model parameters may be updated, how re-training will be validated, and criteria for model acceptance [211].
3. Adherence to Good Machine Learning Practice (GMLP): ensure data representativeness, traceability of versions, bias assessment, and robust documentation of design decisions [212].
4. Integration of AI-specific risk management standards (ISO/IEC 23894 and ISO 14971) to identify and mitigate hazards unique to adaptive algorithms [216].
5. Transparency and explainability appropriate to the model’s risk class, including reporting of uncertainty quantification, population coverage, and human-in-the-loop supervision [213,218].
6. Prospective multicentre validation and real-world performance monitoring to verify model generalizability and detect drift after deployment [211,214].
7. Data governance and security: implement policies for data quality, lineage, anonymization, and federated learning where direct data sharing is restricted [213,218].
8. Interoperability and human-factors design to ensure seamless integration with EHR or PACS systems, minimize workflow burden, and maintain clinician oversight [214,216].

Collectively, these frameworks establish a pathway for translating AI research into safe and ethically compliant clinical tools. By aligning with evolving regulatory guidance, developers and researchers can enhance transparency, trust, and patient safety while facilitating the responsible adoption of adaptive AI systems in medicine.

## 5. Conclusions

Artificial intelligence is rapidly maturing from experimental toolset to practical enabler across multiple biomedical domains. Deep learning architectures, convolutional networks for images, recurrent and transformer models for sequences and text, and graph-based approaches for networked data, have shown strong ability to extract subtle, clinically meaningful signals from imaging, omics, electrophysiology, and behavioral streams. In oncology and nanomedicine, these methods accelerate design and optimization workflows, predict biodistribution and response, and enable richer monitoring using nanoparticle-enhanced imaging and wearable sensors. In cardiology, AI improves automated image quantification, calcium scoring, ECG-based screening and noninvasive functional assessment; in neurology, multimodal models and speech analysis support earlier detection and longitudinal tracking of Parkinson’s and Alzheimer’s disease. Hepatology benefits from automated histopathology, intraoperative assistance, and allocation-model improvements that move toward more personalized transplant prioritization. Despite these advances, implementation barriers remain substantial. Model performance is often contingent on dataset quality and representativeness; heterogeneous acquisition protocols, limited external validation, and cohort-specific biases reduce generalizability. Many high-performing networks retain “black-box” characteristics that complicate clinical trust and regulatory approval, while computational demands and integration challenges limit deployment in routine workflows. Ethical concerns, privacy, fairness, and equitable access, require proactive governance. To translate promise into practice, emphasis should shift to rigorous, prospectively designed multisite validation, harmonized data standards, explainable and uncertainty-aware modeling, and workflows that position AI as an assistive partner to clinicians rather than a replacement. When these technical, clinical, and social requirements are met, AI is positioned to deliver more precise diagnostics, individualized therapies, and scalable monitoring systems that improve outcomes while preserving safety and equity.

## Figures and Tables

**Table 1:** Summary of AI application in medicine.

Topic/Area	Description	Ref.
AI in Medicine	AI enables computers and robots to emulate human behavior, assist in healthcare diagnosis, and perform surgical procedures. Applications include drug development, medical data generation, and disease analysis such as cancer.	[21]
AI-Powered Robotics	AI-driven surgical robots and nanorobots improve precision and efficacy by enabling targeted drug delivery.	[22,23]
AI-Enhanced Soft Robotics	Machine learning–driven soft robotic systems mimic physiological functions for diagnostic and therapeutic applications. Recent advances include ML-enhanced soft robotic platforms inspired by rectal functions to model fecal continence mechanisms and investigate neuromuscular coordination, highlighting the convergence of AI, robotics, and biomedical engineering.	[24]
Machine Learning and Deep Learning in Healthcare	ML and deep learning support clinical diagnostics and treatment decisions. AI-assisted surgical robots are used in procedures like heart valve repair, gynecology, and prostatectomy. Future cancer treatments may rely on unsupervised and reinforcement learning for pattern recognition and strategy optimization.	[25,26,27,28]
AI in Computational Biology and Molecular Medicine	AI contributes to identifying medicinal targets, managing protein interactions, and advancing genetics and molecular medicine.	[29]
Robotic Surgery in Oncology	The review highlights the advantages, challenges, and Indian context of robotic surgery in oncology.	[30]
Case Example: Da Vinci Robotic Surgical System	Yang et al. described a uniportal right upper lobectomy performed with the 4th generation Da Vinci Xi system, demonstrating advanced robotic-assisted surgery capabilities and fast patient recovery.	[31]
Robotic Surgery in Rectal Cancer	Robotic surgery helps overcome limitations of traditional laparoscopy, improving radical operation outcomes. Innovations include the Verb Surgical project and developments in robotic mesorectal excision, lymph node dissection, and AI integration in surgery.	[32]
Computational Methods in Drug Formulation	Computational modeling optimizes drug formulations (e.g., methotrexate nanosuspension) by analyzing molecular interactions and aggregation. Tools like LAMMPS and GROMACS assess nanoparticle behavior. Mehta et al. reviewed these modeling tools, emphasizing their role in personalized medicine and improved therapeutic outcomes.	[33]

**Table 2:** Comparison of AI applications in CT versus MRI for cardiovascular imaging.

Modality	AI Application/Task	Clinical/Research Use	Advantages	Limitations/Challenges	Ref.
CT—Opportunistic risk stratification (DASSi)	AI-based biomarker extraction from echocardiographic + CMR inputs (Digital Aortic Stenosis Severity Index, DASSi)	Screening and follow-up; risk stratification using even handheld devices for opportunistic screening	Enables personalized screening without complex imaging setups; usable on lower-resource platforms	Depends on heterogenous inputs (echo + CMR); needs cross-modality harmonization and validation across populations	[74]
CT—AI screening for valve disease (mitral/aortic)	Automated detection/classification of valve disease severity from imaging and ECG/clinical inputs	Large-scale screening, triage, identification of severe aortic stenosis (AS)	High diagnostic performance (AUCs reported >0.88–0.91 in extreme-spectrum cohorts); enables fast triage	Spectrum and selection bias in training data; model interpretability issues; variable imaging acquisition protocols	[75,76]
CT—Coronary artery calcium scoring (CAC, Agatston) automated	Automated CAC detection and Agatston score estimation from non-contrast/low-dose chest CT or CCTA	Risk stratification for coronary atherosclerosis; population screening (e.g., lung CT cohorts)	High throughput; reduces labor for manual scoring; can be applied opportunistically to lung screening CTs	Image noise, motion or blooming artifacts degrade accuracy; requires robust pre-processing and well-labelled training sets	[77,78,79,80]
CT—CCTA + myocardial analysis for ischemia prediction	Deep learning analysis of left ventricular (LV) myocardium (multiscale CNN + auto-encoding) to predict functionally significant stenosis and stress ischemia	Noninvasive functional assessment adjunct to stenosis grading; improve prediction of ischemia beyond stenosis %	Adds myocardial functional info from standard CCTA; improved discrimination (AUC~0.76 vs. anatomy alone)	Moderate specificity in some reports (e.g., sensitivity 84.6%, specificity 48.4%); method complexity and need for robust validation	[81]
CT—Automated coronary segmentation & classification	DL architectures (EfficientNet, DenseNet201, ResNet101, Xception, MobileNet-v2) for artery segmentation and lesion classification	Automated reporting, quantification of stenosis and plaque characterization	Very high reported metrics in some models (DenseNet201: accuracy 0.90; AUC 0.9694; specificity 0.9833)	Black-box models, potential overfitting to homogeneous datasets; generalizability issues	[71,72]
CT—Lipid/phenogroup clustering for risk prediction (non-imaging input)	Unsupervised ML to derive phenogroups from lipid profiles to predict outcomes in STEMI	Risk stratification and phenotyping for prognosis and personalized management	Reveals biologically meaningful patient subgroups; strong statistical associations with outcomes	Requires large cohorts and external validation; confounding by treatment and comorbidities	[73]
MRI—Automated QA and pre-processing checks	Automated assessment of image quality and slice selection (e.g., ascending vs. descending aorta detection; basal/apical slice identification; motion-artifact detection)	Quality control before downstream analysis; ensures standardized inputs for segmentation and quantification	Reduces manual QC burden; ensures consistent inputs for AI pipelines; lowers inter-scan variability	Models need to handle wide scanning protocols and scanner vendors; edge cases (severe artifacts) may fail	[82,83,84,85]
MRI—Left ventricle (LV) detection and segmentation	CNNs/boundary-regression/regression-based networks for LV identification and segmentation across cardiac cycle	Automated EF calculation, volumetry, mass, wall motion analysis for clinical and research use	Extremely high detection/segmentation accuracy reported (e.g., LV detection success ~99.98%; Dice scores up to ~0.95); much faster than manual tracing	Requires large annotated datasets (tens of thousands of scans); variation across centers; need for robust external validation	[86,87,88,89,90,91]
MRI—Scar quantification and tissue characterization	Deep CNNs for scar volume (late gadolinium enhancement), T1/T2-mapping radiomics, and myocardial tissue feature extraction	Phenotyping for HCM, ischemic scar, fibrosis assessment, prognosis	Enables quantitative, reproducible tissue characterization; radiomics can discriminate diseases (e.g., HCM vs. hypertensive disease)	Radiomics feature reproducibility across scanners and protocols is challenging; requires harmonization and large multisite datasets	[92,93,94]

**Table 5:** Machine Learning in AD Diagnosis and Classification.

Algorithm/Model	Description and Study Findings	Ref.
Support Vector Machine (SVM)	A supervised ML tool used to classify AD and MCI by detecting patterns in labeled imaging data. Widely used in neuroimaging for AD/MCI diagnosis.	[138]
Modular-LASSO Feature Selection (MLFS) + SVM	Zhang et al. developed a hybrid MLFS–SVM method incorporating Fuzzy Bayesian Networks for feature detection in resting-state fMRI, enhancing AD/MCI classification accuracy.	[139]
Radiomics-Based SVM Models	Jiao et al. applied SVM to identify radiomics signatures from tau tracer PET images, achieving higher accuracy (84.8 ± 4.5%) compared to SUVR (73.1 ± 3.6%).	[140,141]
SVM for FDG PET Imaging	Nuvoli et al. used linear SVM on FDG PET imaging for AD/MCI differential diagnosis, reporting 76.23% accuracy based on temporal lobe hypometabolism.	[142]
Emphasis Learning with SVM	Akramifard et al. improved classification performance by repeating key features in smaller datasets (emphasis learning), achieving 98.81% accuracy between AD and normal controls.	[143]
SVM + Graph Theory (fMRI)	Wang et al. combined SVM with graph-based measures for fMRI analysis, yielding 96.80% accuracy in distinguishing AD from healthy controls. Slightly lower accuracy was seen in MCI classification.	[144]
SVM + LASSO (Graph-based fMRI)	Combined SVM and LASSO feature selection provided high accuracy in classifying AD, MCI, and healthy controls, outperforming traditional methods.	[145]
Logistic Regression (LR)	LR explores input–output correlations using a sigmoid curve. Van Loon et al. introduced StaPLR (stacked penalized logistic regression) for multimodal MRI data fusion, achieving a mean AUC of 0.942, outperforming elastic net regression (AUC 0.848).	[146,147]
Decision Tree (DT) and Random Forest (RF)	Supervised ML methods that classify data hierarchically; RF uses multiple DTs to predict outcomes. Widely applied for AD classification.	[145,148,149,150]
Multimodal RF (Aβ PET + sMRI)	Bao et al. demonstrated improved AD classification accuracy using multimodal fusion (AUC = 0.89) compared to single-modality models (AUC = 0.71).	[149]
RF in Feature Selection (Combined with SVM)	Keles et al. used RF as a classifier in combination with optimization algorithms (BABC, BPSO, BGWO, BDE), achieving accuracies of 0.863–0.905.	[151]
Comparison of RF, SVM, MLP, and CNN	Song et al. compared multiple models for AD classification using 63, 29, and 22 features. All models showed high accuracy, but RF exhibited the smallest performance drop (−3.8%), confirming its robustness across feature sets and modalities.	[150]

## Data Availability

No new data were created or analyzed in this study. Data sharing is not applicable.

## Supplementary Materials

The following supporting information can be downloaded at: https://www.mdpi.com/article/10.3390/pharmaceutics17121564/s1, Table S1: PRISMA_2020_checklist.
