# Supplementary material for: Artificial Intelligence in Biomedicine: A Systematic Review from Nanomedicine to Neurology and Hepatology
Source: Pharmaceutics. 2025 Dec 4;17(12):1564. doi: 10.3390/pharmaceutics17121564 (PMC12736181; doi:10.3390/pharmaceutics17121564)
Supplement: Supplementary file 1 [file pharmaceutics-17-01564-s001.zip › pharmaceutics-3992691-supplementary.pdf]

PRISMA 2020 Checklist

Section and Topic	Item #	Checklist item	Location where item is reported
**TITLE**			
Title	1	Identify the report as a systematic review.	Title page – Artificial Intelligence in Biomedicine: An Integrative Review from Nanomedicine to Neurology and Hepatology
**ABSTRACT**			
Abstract	2	See the PRISMA 2020 for Abstracts checklist.	Abstract
**INTRODUCTION**			
Rationale	3	Describe the rationale for the review in the context of existing knowledge.	Introduction (p. 1–2)
Objectives	4	Provide an explicit statement of the objective(s) or question(s) the review addresses.	End of Introduction
**METHODS**			
Eligibility criteria	5	Specify the inclusion and exclusion criteria for the review and how studies were grouped for the syntheses.	Methods, paragraph 2
Information sources	6	Specify all databases, registers, websites, organisations, reference lists and other sources searched or consulted to identify studies. Specify the date when each source was last searched or consulted.	Methods, paragraph 1
Search strategy	7	Present the full search strategies for all databases, registers and websites, including any filters and limits used.	Methods, paragraph 1 (keywords listed)
Selection process	8	Specify the methods used to decide whether a study met the inclusion criteria of the review, including how many reviewers screened each record and each report retrieved, whether they worked independently, and if applicable, details of automation tools used in the process.	Methods, paragraph 3
Data collection process	9	Specify the methods used to collect data from reports, including how many reviewers collected data from each report, whether they worked independently, any processes for obtaining or confirming data from study investigators, and if applicable, details of automation tools used in the process.	Methods, paragraph 4
Data items	10a	List and define all outcomes for which data were sought. Specify whether all results that were compatible with each outcome domain in each study were sought (e.g. for all measures, time points, analyses), and if not, the methods used to decide which results to collect.	Methods, paragraph 4
	10b	List and define all other variables for which data were sought (e.g. participant and intervention characteristics, funding sources). Describe any assumptions made about any missing or unclear information.	Methods, paragraph 4
Study risk of bias assessment	11	Specify the methods used to assess risk of bias in the included studies, including details of the tool(s) used, how many reviewers assessed each study and whether they worked independently, and if applicable, details of automation tools used in the process.	Methods – paragraph on risk of bias/methodological quality assessment (adapted JBI/AMSTAR 2, qualitative consensus-based appraisal, no automation tools).
Effect measures	12	Specify for each outcome the effect measure(s) (e.g. risk ratio, mean difference) used in the synthesis or presentation of results.	Methods – effect measures: no pooled effect measures; study-level metrics (accuracy, sensitivity, specificity, AUC, F1, Dice, etc.) extracted and reported narratively and in tables.
Synthesis methods	13a	Describe the processes used to decide which studies were eligible for each synthesis (e.g. tabulating the study intervention characteristics and comparing against the planned groups for each synthesis (item #5)).	Methods – description of domain-based (nanomedicine, cardiology, neurology, hepatology) and task/modality-based grouping, used to allocate studies to the narrative syntheses and summary tables.

Section and Topic	Item #	Checklist item	Location where item is reported
	13b	Describe any methods required to prepare the data for presentation or synthesis, such as handling of missing summary statistics, or data conversions.	Methods – data preparation: no transformations or imputations; metrics and sample sizes extracted as reported, missing items recorded as ‘not reported’ and not estimated.
	13c	Describe any methods used to tabulate or visually display results of individual studies and syntheses.	Methods (Synthesis of results); Section 3 (Tables 1–5; Figures 1–3).
	13d	Describe any methods used to synthesize results and provide a rationale for the choice(s). If meta-analysis was performed, describe the model(s), method(s) to identify the presence and extent of statistical heterogeneity, and software package(s) used.	Methods (Synthesis of results: narrative, domain-based; no meta-analysis due to heterogeneity).
	13e	Describe any methods used to explore possible causes of heterogeneity among study results (e.g. subgroup analysis, meta-regression).	Methods (no formal statistical exploration of heterogeneity); Section 4. Qualitative Appraisal and Section 4.1 (narrative discussion of heterogeneity sources).
	13f	Describe any sensitivity analyses conducted to assess robustness of the synthesized results.	Methods (no sensitivity analyses; no statistical synthesis performed).
Reporting bias assessment	14	Describe any methods used to assess risk of bias due to missing results in a synthesis (arising from reporting biases).	Methods (reporting bias assessed qualitatively); Section 4. Qualitative Appraisal and Section 4.1 (limitations due to incomplete reporting).
Certainty assessment	15	Describe any methods used to assess certainty (or confidence) in the body of evidence for an outcome.	Methods (qualitative judgement of certainty; no formal GRADE); Section 4. Qualitative Appraisal of Study Quality.
**RESULTS**			
Study selection	16a	Describe the results of the search and selection process, from the number of records identified in the search to the number of studies included in the review, ideally using a flow diagram.	Methods – paragraph describing 295 records identified, 29 duplicates removed, 266 screened, 233 full-texts assessed, 203 included; Figure 2 – PRISMA Flow Diagram of Study Selection Process.
	16b	Cite studies that might appear to meet the inclusion criteria, but which were excluded, and explain why they were excluded.	Not done. Reasons and numbers for exclusions (lack of relevance, non–peer-reviewed, non-English) are reported in Methods and summarized in Figure 2, but no individual excluded studies are cited.
Study characteristics	17	Cite each included study and present its characteristics.	Sections 3. AI in Medicine, 3.1–3.4 (nanomedicine/oncology, cardiology, neurology, hepatology) – narrative description and citation of included studies; Tables 1–5 summarise topic/area, data type, AI task, and key references.
Risk of bias in studies	18	Present assessments of risk of bias for each included study.	Section 4. Qualitative Appraisal of Study Quality – qualitative assessment of methodological quality and potential risk of bias across studies (clarity of objectives, data validity, validation methods, reporting, reproducibility); no individual

Section and Topic	Item #	Checklist item	Location where item is reported
			study-level RoB table is presented.
Results of individual studies	19	For all outcomes, present, for each study: (a) summary statistics for each group (where appropriate) and (b) an effect estimate and its precision (e.g. confidence/credible interval), ideally using structured tables or plots.	Sections 3.1–3.4 – individual study results presented narratively with reported metrics for selected key studies; Tables 1–5 summarise applications and some performance statistics where available. No pooled effect estimates or confidence intervals are calculated for all included studies.
Results of syntheses	20a	For each synthesis, briefly summarise the characteristics and risk of bias among contributing studies.	Sections 3.1–3.4 – domain-based syntheses (nanomedicine/oncology, cardiology, neurology, hepatology) summarising study characteristics, data modalities, AI approaches and main findings; Section 4 and Section 4.1 – cross-domain synthesis of methodological quality, common biases (data heterogeneity, incomplete reporting, limited external validation) and strengths.
	20b	Present results of all statistical syntheses conducted. If meta-analysis was done, present for each the summary estimate and its precision (e.g. confidence/credible interval) and measures of statistical heterogeneity. If comparing groups, describe the direction of the effect.	Not applicable. No statistical syntheses or meta-analyses were performed; results are synthesised narratively by domain (Sections 3.1–3.4).
	20c	Present results of all investigations of possible causes of heterogeneity among study results.	Not applicable. No formal statistical investigation of heterogeneity was conducted. Potential sources of heterogeneity (datasets, imaging protocols, model types, validation strategies) are discussed qualitatively in Section 4 and Section 4.1.
	20d	Present results of all sensitivity analyses conducted to assess the robustness of the synthesized results.	Not applicable. No sensitivity analyses were conducted because no quantitative syntheses were performed.
Reporting biases	21	Present assessments of risk of bias due to missing results (arising from reporting biases) for each synthesis assessed.	No formal statistical assessment of reporting bias was undertaken. Potential risk of bias due to incomplete or selective reporting is described qualitatively in. Methods
Certainty of evidence	22	Present assessments of certainty (or confidence) in the body of evidence for each outcome assessed.	No formal certainty framework was used.
**DISCUSSION**			
Discussion	23a	Provide a general interpretation of the results in the context of other evidence.	Section 4.1. Advantages, Limitations, and Mitigation Strategies of Artificial Intelligence in Biomedicine, and Section 5. Conclusions – overall interpretation of the domain-specific findings and cross-domain trends, discussed in relation to current AI research and clinical translation.

Section and Topic	Item #	Checklist item	Location where item is reported
	23b	Discuss any limitations of the evidence included in the review.	Section 4. Qualitative Appraisal of Study Quality and Section 4.1 – discussion of limitations of the included evidence (data heterogeneity, incomplete reporting, lack of standardized acquisition and reporting, limited external and prospective multicentre validation, cohort bias, ethical/equity concerns).
	23c	Discuss any limitations of the review processes used.	Section 4. Qualitative Appraisal of Study Quality – narrative discussion of review-level limitations, including absence of formal numeric risk-of-bias scoring, no meta-analysis, reliance on narrative synthesis due to heterogeneity, and incomplete reporting in primary studies that constrained comparisons.
	23d	Discuss implications of the results for practice, policy, and future research.	Section 4.1. Advantages, Limitations, and Mitigation Strategies…, Section 4.2. Regulatory and Governance Context of AI in Healthcare (2024–2025), and Section 5. Conclusions – implications for clinical practice, regulatory and governance frameworks, and priorities for future research (standardization, explainable and uncertainty-aware models, multicentre validation, ethical and equity safeguards).
**OTHER INFORMATION**			
Registration and protocol	24a	Provide registration information for the review, including register name and registration number, or state that the review was not registered.	Section 2. Methods – statement that the review was conducted according to PRISMA 2020 and registered on the Open Science Framework (OSF), with registration link https://osf.io/m4fq2.
	24b	Indicate where the review protocol can be accessed, or state that a protocol was not prepared.	Section 2. Methods – OSF registration and protocol access information ("The full protocol and metadata are available at https://osf.io/m4fq2, accessed on 10 November 2025").
	24c	Describe and explain any amendments to information provided at registration or in the protocol.	Not reported. No amendments to the registered protocol are described in the manuscript.
Support	25	Describe sources of financial or non-financial support for the review, and the role of the funders or sponsors in the review.	Funding statement – The Article Processing Charges were funded by the University of Medicine and Pharmacy of Craiova, Romania. The funder had no role in the design, conduct, analysis, or reporting of the review.
Competing interests	26	Declare any competing interests of review authors.	Conflicts of Interest section – "The authors declare no conflicts of interest."
Availability of	27	Report which of the following are publicly available and where they can be found: template data collection	Data Availability Statement – "No new data were

PRISMA 2020 Checklist

Section and Topic	Item #	Checklist item	Location where item is reported
data, code and other materials		forms; data extracted from included studies; data used for all analyses; analytic code; any other materials used in the review.	created or analyzed in this study. Data sharing is not applicable." Protocol and metadata for the review are publicly available via OSF (Section 2. Methods; https://osf.io/m4fq2). No additional data extraction files, analytic code, or templates are reported as publicly available.

From: Page MJ, McKenzie JE, Bossuyt PM, Boutron I, Hoffmann TC, Mulrow CD, et al. The PRISMA 2020 statement: an updated guideline for reporting systematic reviews. BMJ 2021;372:n71. doi: 10.1136/bmj.n71. This work is licensed under CC BY 4.0. To view a copy of this license, visit https://creativecommons.org/licenses/by/4.0/
